# Biosynthesis of butyrate from methanol and carbon monoxide by recombinant *Acetobacterium woodii*

**DOI:** 10.1007/s10123-022-00234-z

**Published:** 2022-02-18

**Authors:** Nilanjan Pal Chowdhury, Dennis Litty, Volker Müller

**Affiliations:** Department of Molecular Microbiology & Bioenergetics, Institute of Molecular Biosciences, Johann Wolfgang Goethe University, Max-von-Laue Str. 9, 60438 Frankfurt, Germany

**Keywords:** Acetogen, Butyrate, Bioengineering, Wood-Ljungdahl pathway, Acetogenesis, Carbon cycling

## Abstract

Methanol is one of the most widely produced organic substrates from syngas and can serve as a bio-feedstock to cultivate acetogenic bacteria which allows a major contribution to reducing greenhouse gas. *Acetobacterium woodii* is one of the very few acetogens that can utilize methanol to produce acetate as sole product. Since *A. woodii* is genetically tractable, it is an interesting candidate to introduce recombinant pathways for production of bio-commodities from methanol. In this study, we introduced the butyrate production operon from a related acetogen, *Eubacterium callanderi* KIST612, into *A. woodii* and show a stable production of butyrate from methanol. This study also reveals how butyrate production by recombinant *A. woodii* strains can be enhanced with addition of electrons in the form of carbon monoxide. Our results not only show a stable expression system of non-native enzymes in *A. woodii* but also increase in the product spectrum of *A. woodii* to compounds with higher economic value.

**Supplementary Information:**

The online version contains supplementary material available at 10.1007/s10123-022-00234-z.

## Introduction

In times of global warming, there is an increasing need to reduce CO_2_ emissions into the atmosphere. Therefore, petroleum-based technologies for the production of added-value compounds need to be replaced by sustainable biotechnological approaches. Various bacteria and archaea are known to convert CO_2_ but strictly anaerobic, acetogenic bacteria have gained much interest in recent times for they grow in the dark in the absence of oxygen, are easy to cultivate, and are metabolically very diverse (Drake 1994; Müller 2003; Drake et al. 2008). They can grow lithotrophically on H_2_ + CO_2_, some also on syngas (H_2_ + CO_2_ + CO), on C1 compounds such as formate or methanol but also organotrophically on various sugars, carbonic acids, aldehyde, primary, and secondary alcohols (Drake et al. 1997; Schuchmann and Müller 2016). Many of these metabolic pathways go along with the fixation of CO_2_. For example, acetogenesis from methanol according to:

1 $$4 {\mathrm{CH}}_{3}\mathrm{OH}+2 {\mathrm{CO}}_{2}\leftrightarrow 3 {\mathrm{CH}}_{3}\mathrm{COOH}+{\mathrm{H}}_{2}\mathrm{O}$$

requires half a mol of CO_2_ per mol of methanol converted.

Methanol is a promising feedstock for acetogens and it can be produced from syngas chemically and then used to feed acetogens without the problems inherent to gas fermentation (Cotton et al. 2019; Satanowski und Bar-Even 2020; Kremp und Müller 2021). Butyrate is such an added value compound that is the starter molecule for butanol production, a biofuel with a much higher energy density than ethanol and some acetogens like *Clostridium carboxidivorans*, *Clostridium drakei* (Liou et al. 2005), *Eubacterium callanderi* KIST612 (Chang et al. 1997; Litty and Müller 2021), and few intestinal acetogens like *Butyvibrio crossotus* and *Eubacterium rectale* (van den Abbeele et al. 2013) have been shown before to produce butyrate naturally. Butyrate was also produced from syngas in co-culture of *Clostridium kluyveri* and *Clostridium autoethanogenum* (Diender et al. 2016). Here, we have metabolically engineered one of the most robust and well-studied acetogens, *Acetobacterium woodii*, as a new production platform for butyrate formation from fructose or methanol.

## Results

### Generation of recombinant A. woodii

**Fig. 1:**
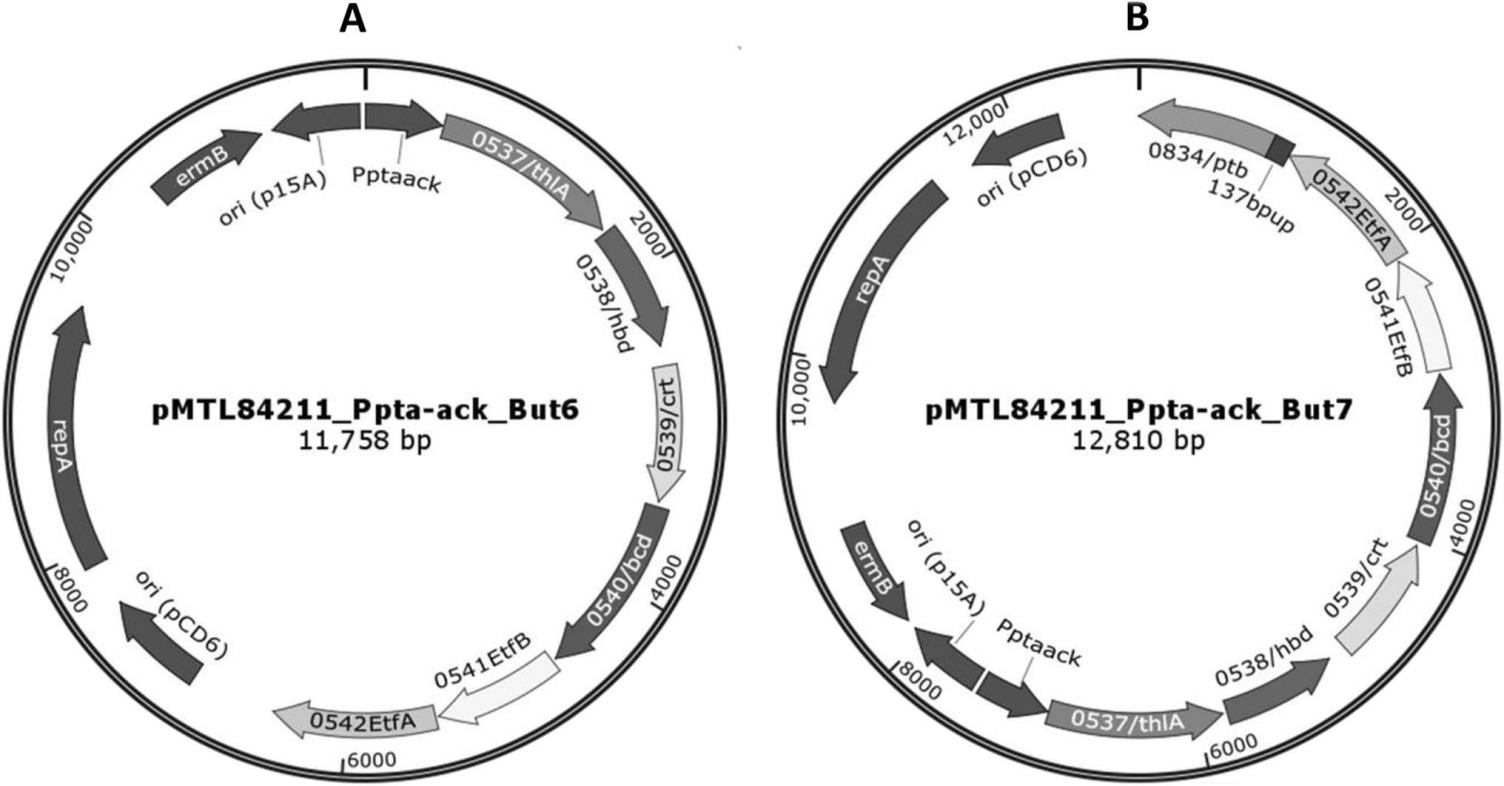
Physical map of plasmids containing genes of recombinant butyrate pathway. The constructed expression plasmid pMTL84211Ack_NP_But6 (**A**) and plasmid pMTL84211Ack_NP_But7 (**B**) harboring six and seven genes, respectively, of butyrate synthetic pathway used to transform WT *A. woodii*. Key: *thlA*, thiolase; *hbd*, hydroxybutyryl-CoA dehydrogenase; *crt*, crotonase; *bcd/etfBA*, bifurcating butyryl-CoA dehydrogenase complex; *ptb*, phosphotransbutyrylase; 137 bpup, upstream region of the ptb gene; pCD6, replicon of *C. difficile* plasmid pD6; repA, gene encoding replicon protein; traJ, transfer function of the RP4 oriT region; ermB, the macrolide-lincosamide-streptogramin B antibiotic resistance gene of plasmid pAMß1, Ppta-ack, the promoter of the *C. ljungdahlii* gene encoding phosphotransacetylase-acetate kinase gene

To generate a recombinant *A. woodii* strain able to produce butyrate, we chose to clone the butyrate production genes from *E. callanderi* KIST612, an acetogen known to produce butyrate naturally. *E. callanderi* KIST612 harbors the genes encoding thiolase (*thlA*, ELI_0537), 3-hydroxybutyryl-CoA dehydrogenase (*hbd*, ELI_0538), crotonase (*crt*, ELI_0539), and the electron bifurcating butyryl-CoA dehydrogenase complex (*bcd/etfAB*, ELI_0540-0542). While the genes of the butyrate pathway are clustered in a single gene cluster, the phosphobutyryl transferase (*ptb*, ELI_0834) is encoded elsewhere in the genome. Unfortunately, there is no annotated butyrate kinase gene in the genome. First, we cloned the genes *thlA*, *hbd*, *crt*, and *bcd/etfAB* of the butyrate cluster in plasmid pMTL84211 (Purdy et al. 2002; Heap et al. 2007) under the control of *pta-ack* (phosphotransacetylase-acetate kinase) promoter from *Clostridium ljungdahlii* giving rise to the plasmid pMTL84211pAck_NP_But6. A second plasmid was also created which contained the *Ptb* gene along with its 137 bp upstream region forming the plasmid pMTL84211pAck_NP_But7 (Fig. 1).

**Fig. 2:**
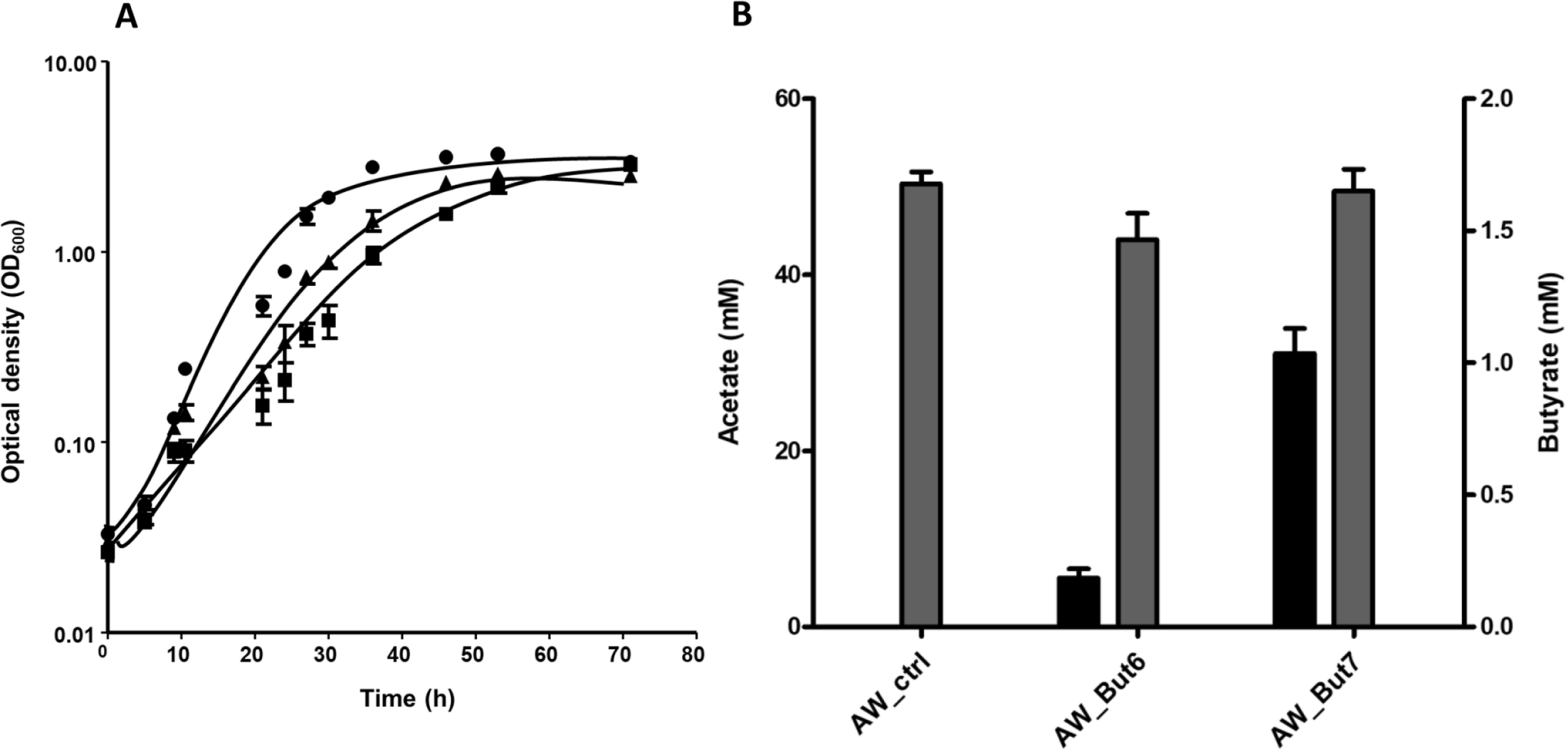
Growth of recombinant *A. woodii* strains on 20 mM fructose. **A** Pre-adapted recombinant *A. woodii* AW_ctrl (black circle), AW_But6 (black square), and AW_But7 (black up-pointing triangle) were grown on 20 mM fructose under a N_2_/CO_2_ [80/20% (v/v)] atmosphere. The cultures were cultivated at 30°C and growth experiments were performed in 50 ml complex media. **B** Determination of acetate and butyrate at end of fructose fermentation; butyrate (black) and acetate (gray). Each data point indicates a mean ± SEM; *n* = 2 independent experiments

The plasmids were transferred into *A. woodii* and the recombinants grew well on fructose with doubling times of 9.8 h (Aw_But6 strain) and 9 h (Aw_But7 strain) to similar final optical densities (Fig. 2). Each of these strains produced acetate, as expected, but Aw_But6 and Aw_But7 also produced butyrate. In Aw_But6, the yield was low but clearly above the control and the yield was increased by 400% up to a concentration of 1.5 mM in Aw_But7 (Fig. 2). In total, the carbon recovery was 78–83%, not accounting for CO_2_.

**Fig. 3:**
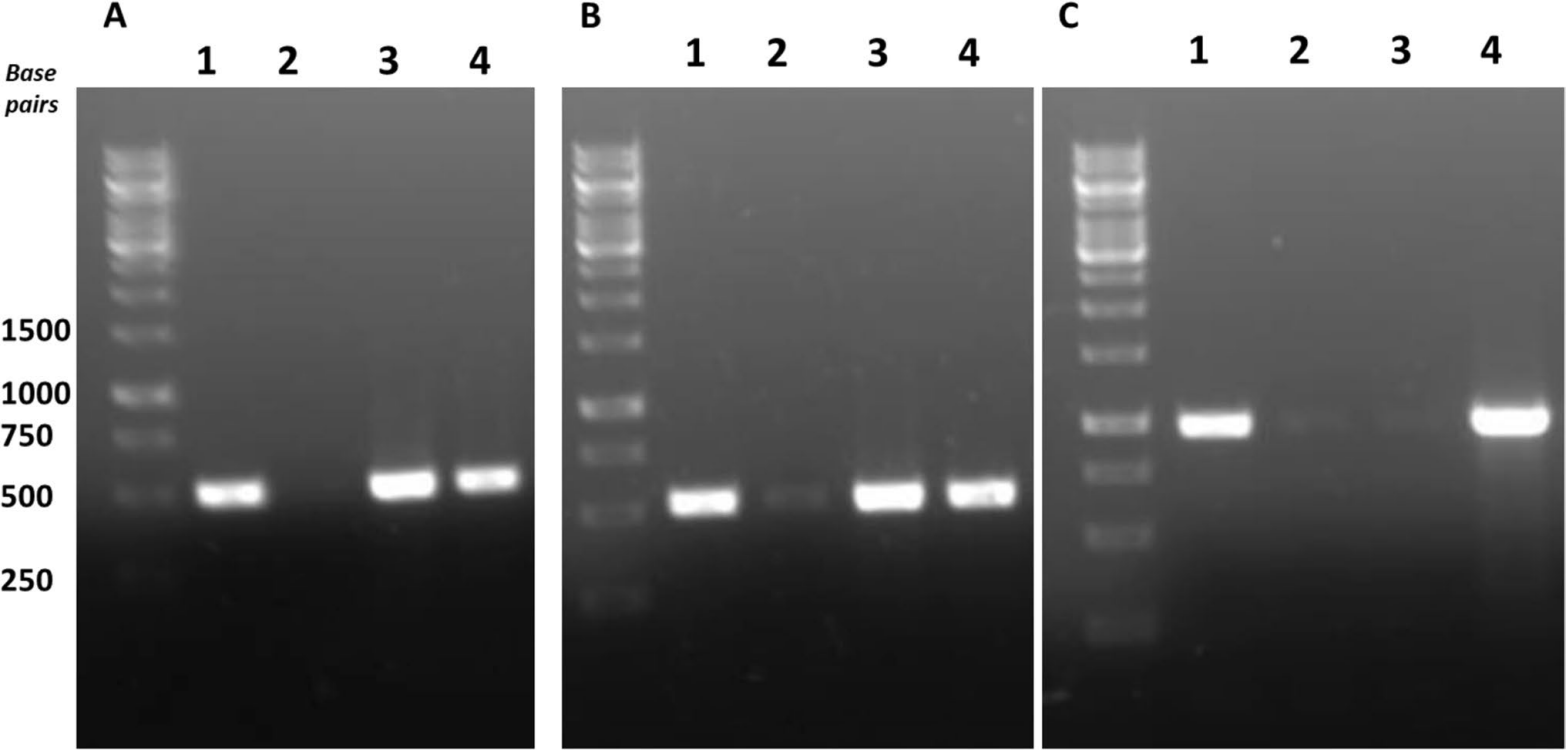
Gene expression analysis of the synthetic butyrate pathway genes in *Acetobacterium woodii*. The transcript abundance of thiolase (**A**), hydroxybutyryl-CoA dehydrogenase (**B**), and phosphate butyryltransferase (**C**) were analyzed with semi-quantitive PCR (24 cycles) with cDNA as template. Chromosomal DNA from *E*. *callanderi* was used as control. Lane 1, *E. callanderi* chromosomal DNA; lane 2, cDNA from WT *A. woodii*; lane 3, cDNA from Aw_But6; lane 4, cDNA from Aw_But7

The increased production of butyrate in Aw_But7 argued for the *ptb* gene being transcribed in addition resulting in an active phosphobutyryl transferase leading to the production of butyryl phosphate which is further dephosphorylated to butyrate. Indeed, this was observed; genes encoding butyrate production like *thlA* and *hbd* were expressed in *A. woodii* during growth on fructose in Aw_But6 and Aw_But7 strain and in addition, *ptb* was also transcribed in Aw_But7 strain (Fig. 3). Apparently, co-transcription of *ptb* led to higher butyrate yields. Although butyrate kinases are not annotated in the genomes of *A*. *woodii* or *E*. *callanderi*, cell-free extract of fructose-grown cells of *A*. *woodii* and *E*. *callanderi* catalyzed butyrate kinase activity with 0.43 and 0.57 U/mg, respectively. Acetate kinase activity was sevenfold higher in *A*. *woodii* (3.1 U/mg) and twice as high in *E*. *callanderi* (1.38 U/mg). Apparently, butyrate kinase activity is present in wild type *A*. *woodii* and *E*. *callanderi*.

### Butyrate production is carbon source dependent

**Fig. 4:**
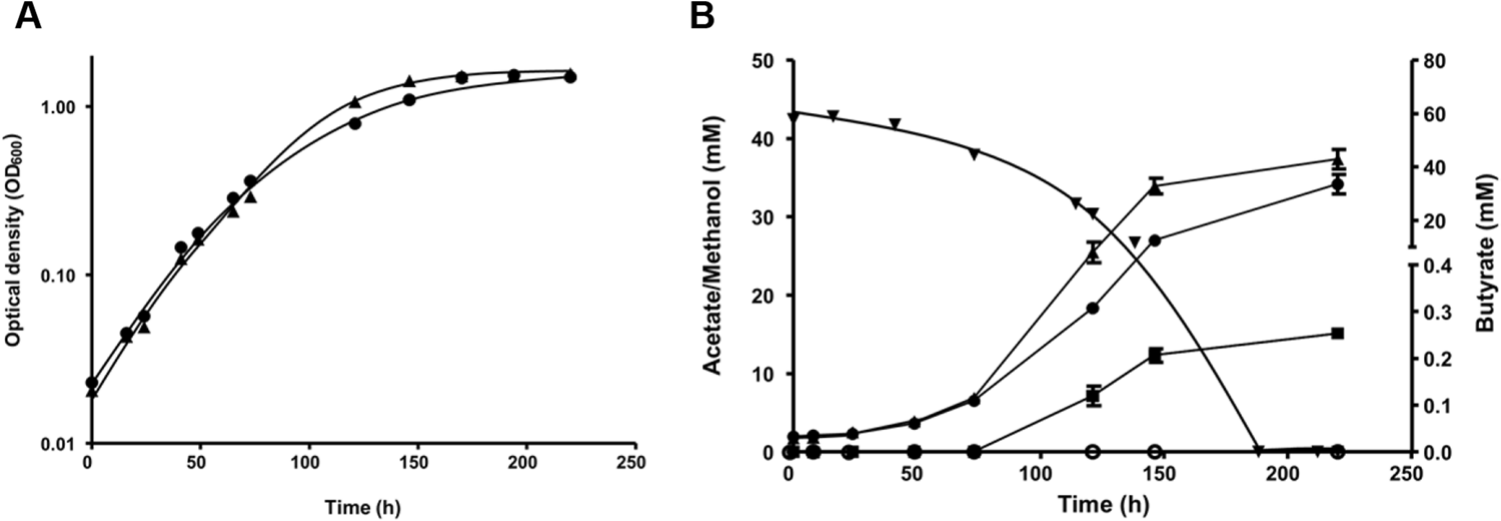
Growth of recombinant *A. woodii* Aw_But6 and Aw_But7 on 60 mM methanol and butyrate formation. **A** Methanol-adapted recombinant *A. woodii* Aw_But6 (black circle) and Aw_But7 (black up-pointing triangle) were grown on 60 mM methanol under a N_2_/CO_2_ [80/20% (v/v)] atmosphere. The cultures were cultivated at 30°C and growth experiments were performed in 500 ml complex media and optical density determined. **B** Concentrations of methanol (black down-pointing triangle), acetate (Aw_But6, black up-pointing triangle; Aw_But7, black circle), and butyrate (Aw_But6, open circle; Aw_But7, black square) were measured as described in “Materials and methods,” and OD_600_ was determined photometrically. All data points are mean ± SEM; *N* = 2 independent experiments

As shown above, butyrate was produced during growth on fructose as carbon source. *A. woodii* also grows lithotrophically on H_2_ + CO_2_, but butyrate was not detected under these conditions in neither Aw_But6 nor Aw_But7 strains (data not shown). Like the wild type, Aw_But6 and Aw_But7 grew well on methanol, although the doubling time was increased (24 h) compared to the wild type (16 h). Methanol consumption was accompanied by the formation of acetate and a total of around 38 mM of acetate was formed from 60 mM methanol, corresponding to an acetate methanol ratio of 0.63. Interestingly, parallel to the formation of acetate, butyrate production started in Aw_But7 strain at the mid exponential growth phase and reached a maximum of 0.25 mM in the stationary phase (Fig. 4). Butyrate formation could not be detected in Aw_But6 strain.

### Butyrate production in non-growing cells

**Fig. 5:**
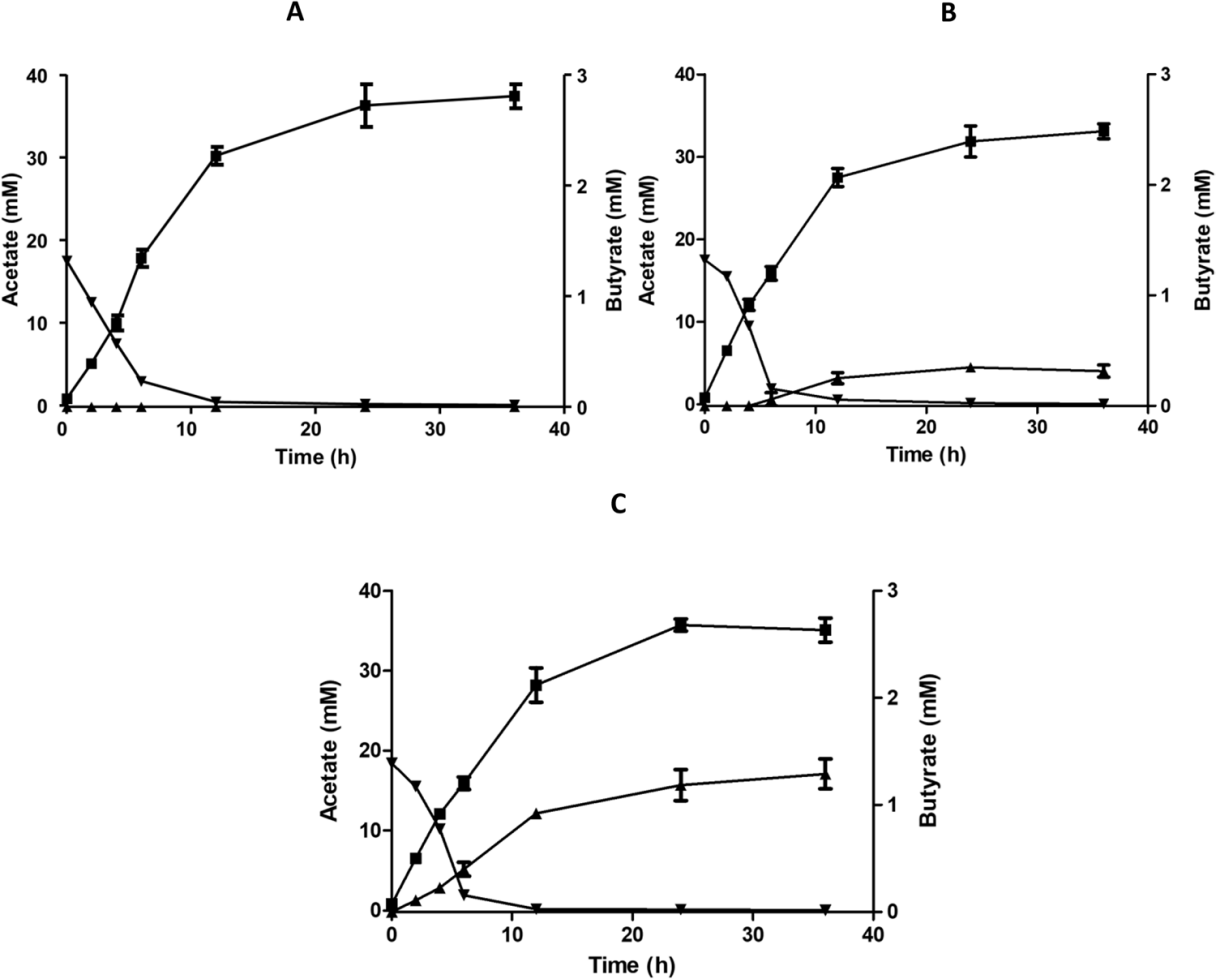
Fructose uptake and conversion into acetate and butyrate by resting cells of recombinant *A. woodii* strains. Recombinant *A. woodii* strains were grown on 20 mM fructose as described in “Materials and methods” and harvested in the late exponential growth phase. The cells were washed twice and resuspended to a final protein concentration of 1 mg ml^−1^ in 10 ml imidazole buffer (50 mM imidazole, 20 mM NaCl, 20 mM KCl, 20 mM MgSO_4_, 60 mM KHCO_3_, pH 7.0) in 115-ml serum bottles under a N_2_/CO_2_ atmosphere (80:20 [v/v]). A total of 20 mM fructose was given to cell suspension of Aw_ctrl (**A**), Aw_But6 (**B**), and Aw_But7 (**C**). Samples were drawn at different time points for quantification of fructose (black down-pointing triangle), acetate (black square), and butyrate (black up-pointing triangle). All data points are mean ± SEM; *N* = 2 independent experiments

Non-growing, resting cells have the advantage of not losing carbon to biosynthetic pathways. Therefore, we analyzed butyrate formation in resting cells. Cells grown on fructose and resuspended in buffer immediately started to produce acetate from fructose. Aw_ctrl strain, not carrying the butyrate synthetic genes, produced 37 mM acetate from 17 mM fructose. As expected, butyrate was not produced. Interestingly, in the recombinant strain, unlike in growing condition, fructose consumed to acetate formed was lower (1:2.1) compared to growing cells (~ 1:2.5). Aw_But6 produced less acetate (36 mM), but when production started to reach the plateau, butyrate production started. Finally, 0.3 mM butyrate was produced, similar to observed under growing conditions. In comparison to Aw_But6 strain, Aw_But7 carrying *ptb*, in addition, produced similar amounts of acetate (35 mM) but butyrate formation was increased drastically by 400% to a final concentration of 1.2 mM (Fig. 5).

**Fig. 6:**
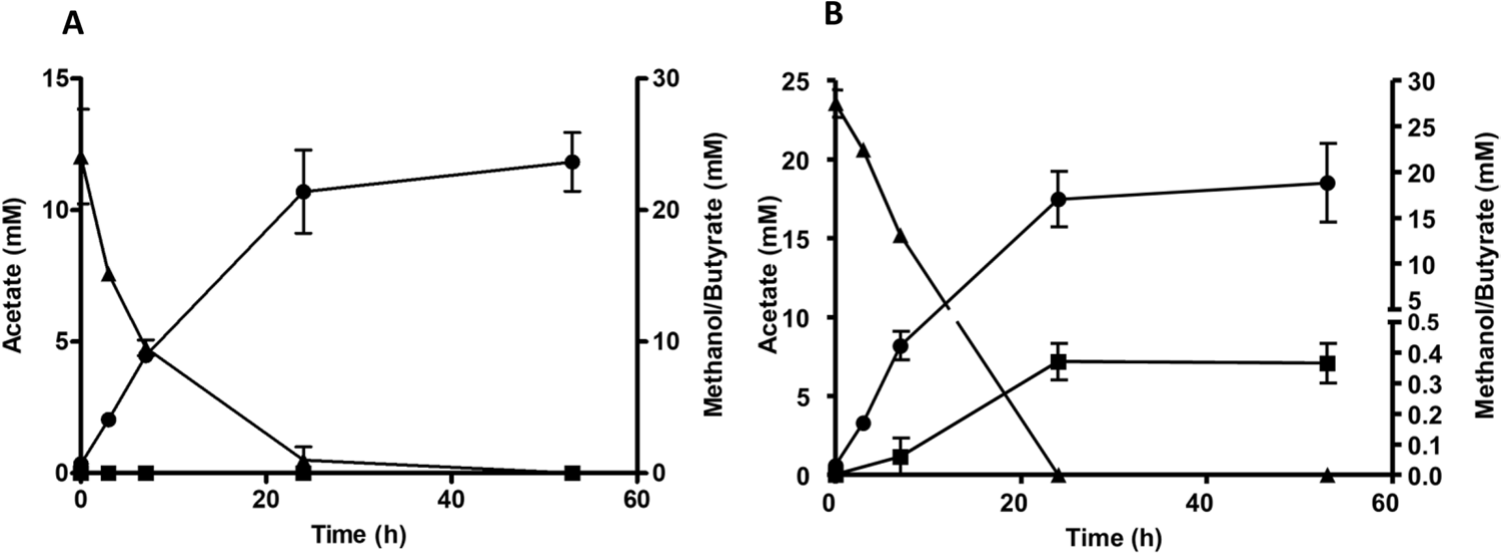
Methanol uptake and conversion into acetate and butyrate by resting cells of recombinant *A. woodii*. *A. woodii* Aw_But7 was grown on 60 mM methanol as described in the “Materials and methods” section and harvested in the late exponential growth phase. The cells were washed twice and resuspended to a final protein concentration of 0.8 mg ml^−1^ in 10 ml imidazole buffer (50 mM imidazole, 20 mM NaCl, 20 mM KCl, 20 mM MgSO_4_, 60 mM KHCO_3_, pH 7.0) in 115-ml serum bottles. The experiments were performed under a N_2_/CO_2_ atmosphere (80:20) (**A**) additional 10% CO (**B**). Samples were drawn at different time points for quantification of acetate (black circle), butyrate (black square), and methanol (black up-pointing triangle) by gas chromatography. All data points are mean ± SEM; *N* = 2 independent experiments

Cells of Aw_But7, grown on methanol and resuspended in buffer, also metabolized methanol but in contrast to growing cells, only acetate was produced and butyrate could not be detected. However, when carbon monoxide (10% v/v) was added to the headspace, acetate formation increased and concomitantly butyrate was produced up to a maximum concentration of 0.35 mM (Fig. 6).

## Discussion and conclusion

The global-interest in reducing levels of CO_2_ and increase carbon re-cycling led the scientific community to find several effective methods to fix CO_2_ artificially and also use microorganisms to convert CO_2_ into biochemicals. In recent years, a lot of success has been achieved where acetogens like *Clostridium autoethanogenum* (Köpke et al. 2014; Liew et al. 2016a, b; Heffernan et al. 2020), *Clostridium ljungdahlii* (Ueki et al. 2014), and *Moorella thermoacetica* (Kita et al. 2013) had been engineered with genes from other clostridia to utilize H_2_ + CO_2_ or syngas to produce higher carbon chain biochemicals. In a proof-of-concept study, it was shown that *A*. *woodii* can produce acetone under autotrophic conditions when the thiolase, CoA transferase, and acetoacetate decarboxylase genes from *Clostridium acetobutylicum* were expressed (Hoffmeister et al. 2016). While most biochemical production pathways are energy invasive, in a very recent study, recombinant *A. woodii* (Beck et al. 2020) was shown to conserve additional energy when the arginine deiminase pathway from *C. autoethanogenum* was heterologusly produced in *A. woodii*. The production of longer carbon-chain compounds in *A. woodii* would also require additional supply of energy and indeed this approach will find its merit.

Here in this study, we rather ask a very simple question. Can we can transfer metabolic pathways within related acetogens and induce them to produce a chemical that the other does not produce? It has been reported before that under certain circumstances, *E. callanderi* KIST612 naturally produces butyrate along with acetate as sole end products (Jeong et al. 2015; Dietrich et al. 2021; Litty and Müller 2021). The results presented here clearly demonstrate that transferring the butyrate formation pathway in a related acetogen like *A. woodii* that does not produce butyrate naturally leads to production of butyrate in the recombinant *A. woodii* strains. However, the maximal amount of butyrate produced during growth on 60 mM methanol was rather low (0.25 mM) in comparison to *E*. *callanderi* KIST612 (3.7 mM butyrate on 20 mM methanol). While the butyrate formation pathway requires the eight proteins thiolase, hydroxybutyryl-CoA dehydrogenase, crotonase, butyryl-CoA dehydrogenase/electron transferring flavoprotein complex, phosphotransbutyrylase, and butyrate kinase, introduction of only seven of the protein encoding genes into *A. woodii* led to continued formation of butyrate from both C6 and C1 compounds. Though the terminal gene of the butyrate pathway encoding for a butyrate kinase was missing, it is likely that the highly active acetate kinase (*ack*, Awo_c21260) synthesizes butyrate from butyryl-phosphate in recombinant *A. woodii* (Eden und Fuchs 1983; Schuchmann and Müller 2014). Indeed, cell-free extract of *A*. *woodii* showed butyrate kinase activity. Whether this is done by the acetate kinase or by an unknown butyrate kinase remains to be established. The same is true for *E*. *callanderi*. It should also be noted that the acetate kinase from *Methanosarcina thermophila*, which is 60% identical to the acetate kinase from *A*. *woodii*, can utilize butyrate, although with a 50-fold higher k_m_ value (Ingram-Smith et al. 2005).

**Fig. 7:**
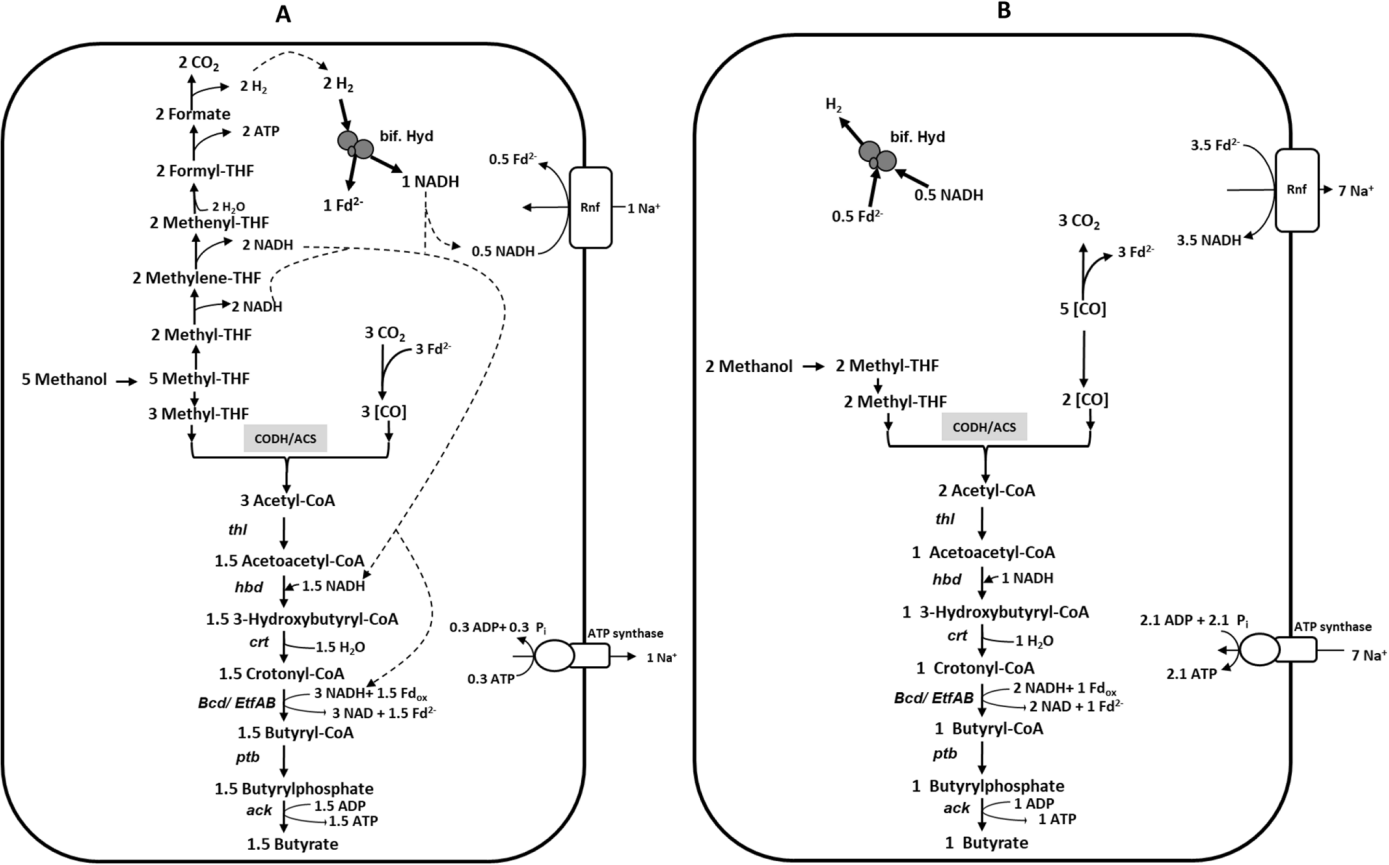
Model of butyrate formation from methanol (**A**) or methanol + CO (**B**). Carbon and probable electron flow in methanol metabolism of butyrate producing recombinant *A. woodii*; for explanation, see text. The stoichiometry of the ATPase is 3.3 Na^+^/ATP (Matthies et al. 2014), and the stoichiometry of the Rnf complex is assumed to be 2 Na^+^/2 electrons. Abbreviations: CODH/ACS, CO dehydrogenase/acetyl-CoA synthase complex; bif. Hyd, electron bifurcating hydrogenase; Rnf, Rnf complex; *thlA*, thiolase; *hbd*, hydroxybutyryl-CoA dehydrogenase; *crt*, crotonase; *bcd/etfBA*, bifurcating butyryl-CoA dehydrogenase complex; *ptb*, phosphotransbutyrylase

Also, we could show that recombinant *A. woodii* could produce a four-carbon product (C4) from a C1 compound like methanol. The metabolism of methanol in recombinant *A. woodii* likely involves the methanol-specific methyltransferase system (Kremp et al. 2018; Kremp and Müller 2021) to generate methyl-THF which further condenses with an incoming CO using CODH/ACS enzyme to generate the central metabolic intermediate, acetyl-CoA. In recombinant strains, acetyl-CoA is further metabolized downstream to produce acetate or butyrate and conserve energy. The anaerobic utilization of methanol via the WLP provides NADH but needs an input of reduced ferredoxin for reduction of CO_2_ to CO (Fig. 7A). Reduced ferredoxin is generated from NADH by reverse electron transport catalyzed by the Rnf complex, energized by ATP hydrolysis (Kremp and Müller 2021). However, the production of butyrate from methanol requires NADH (Song et al. 2017) by hydroxybutyryl-CoA dehydrogenase and the electron bifurcating Bcd/EtfAB complex (Buckel und Thauer 2013; Jeong et al. 2015; Katsyv and Müller 2020) (Fig. 7A), which are generated by methanol oxidation. Importantly, the electron bifurcating butyryl-CoA dehydrogenase provides reduced ferredoxin, the fuel for electron transport phosphorylation thus increasing the ATP yield. In theory, from 5 methanol and 1 CO_2_, 1.5 butyrate could be produced leading to an ATP yield of 0.64 mol ATP/mol methanol consumed according to Eq. 2 (Fig. 7A):

2 $$5 {\mathrm{CH}}_{3}\mathrm{OH}+1 {\mathrm{CO}}_{2}+3.2\mathrm{ ADP}+3.2\mathrm{ Pi }\to 1.5 {\mathrm{CH}}_{3}{\mathrm{C}}_{2}{\mathrm{H}}_{4}\mathrm{COOH}+4 {\mathrm{H}}_{2}\mathrm{O}+3.2\mathrm{ ATP}$$

In recombinant strains, the effective utilization of NADH is supposed to shift the acetate:butyrate ratio in favor of butyrate when cells are grown on methanol + CO_2_. This was true for Aw_But7 strain during growth on methanol (60 mM). However, the recovery of carbon in form of butyrate was relatively low in comparison to acetate, which is attributed either by the missing butyrate kinase or electron imbalance. In such a scenario, during cell suspension assays, with reduced electron pressure in form of lower concentration of methanol (20 mM), Aw_But7 strain did not synthesize butyrate.

Our results show that this electron imbalance could be partially surpassed by introduction of electron rich CO gas into the gaseous phase which significantly enhanced butyrate formation in the recombinant strain. In theory, butyrogenesis from methanol + CO is simple and involves condensation of 2 methanol with 2 CO leading to the formation of 1 butyrate. Also, owing to the reduced ferredoxin generated by CO oxidation, in this modular branch, an increase in the energy efficiency by almost 140% can be obtained with an ATP yield of 1.55 mol ATP/mol methanol consumed according to Eq. 3 (Fig. 7B):

3 $$2 {\mathrm{CH}}_{3}\mathrm{OH}+5\mathrm{ CO}+3.1\mathrm{ ADP}+3.1\mathrm{ Pi}\to 1 {\mathrm{CH}}_{3}{\mathrm{C}}_{2}{\mathrm{H}}_{4}\mathrm{COOH}+3 {\mathrm{CO}}_{2}+1 {\mathrm{H}}_{2}+3.1\mathrm{ ATP}$$

However, in this case, addition of CO also increased acetate formation and this stresses the necessity of tailoring the pathway by specific genetic deletion to redirect the carbon flux towards butyrate instead of acetate. One such approach would be to delete the acetate kinase or phosphotransacetylase gene and introduce a clostridial butyrate kinase to complete the butyrate synthetic pathway. Indeed, it was reported that deletion of *pta* in *C. ljungdahlii* led to a decrease in acetate production by > 80% and improved ethanol formation via aldehyde ferredoxin oxidoreductase (*AOR*) (Lo et al. 2020). We could imagine that a similar deletion in *A. woodii* would lead to an accumulation of acetyl-CoA and in the presence of a recombinant butyrate pathway, the carbon flux would also be pushed towards the ATP generating butyrate synthesis. Finally, this study shows that *A. woodii* can be made to produce butyrate from methanol or methanol + CO which makes methanol a promising feedstock for an alternative bioeconomy using acetogens as biocatalyst.

## Materials and methods

### Cultivation of A. woodii and E. callanderi KIST612

*A. woodii* DSMZ 1030 and transformants were cultivated under strict anoxic conditions at 30°C in carbonate buffered complex medium. The medium was prepared as described previously (Heise et al. 1989). Fructose (20 mM), methanol (60 mM), or H_2_ + CO_2_ (80:20 [v/v]) served as a sole carbon and energy source for butyrate production studies. Transformed *A. woodii* cells harboring butyrate pathway genes were first selected on Heise media containing 20 mM fructose and 15 µg/ml erythromycin. *E. callanderi* KIST612 was cultivated at 37°C in anoxic carbonate-buffered basal medium (CBBM) (Chang et al. 1999) with glucose under a N_2_/CO_2_ (80/20% [v/v]) atmosphere. Growth was followed by measuring the optical density at 600 nm. All growth experiments were performed in 115-ml serum flasks containing 50 ml of media.

### Construction of plasmids for butyrate production

For the construction of a synthetic pathway, the genes necessary for synthesis of butyrate in *E. callanderi* KIST612 were selected and amplified by PCR. The genes clustered in the butyrate operon, ELI_0537 – ELI_0542, consisting of thiolase (*thlA*), 3-hydroxybutyryl-CoA dehydrogenase *(hbd*), crotonase (*crt*), butyryl-CoA dehydrogenase (*bcd*), and two subunits of electron transferring flavoprotein (*EtfA/B)* were amplified and subcloned into pMTL84211 backbone (Purdy et al. 2002) together (upstream) with PCR-amplified P_*pta-ack*_ promoter from *C. ljungdahlii*. The resulting plasmid was called pMTL84211Ack_NP_But6. Furthermore, the phosphobutyryl transferase (*ptb*, ELI_0834) with its 137 bp upstream region was also amplified by PCR and subcloned into the pMTL84211_6kb plasmid, downstream of *etfA* (ELI_0542). The resulting plasmid had 7 genes (*thl*, *hbd*, *crt*, *bcd/EtfAB*, and *ptb*) of the butyrate synthetic pathway and was called pMTL84211Ack_NP_But7. A control plasmid was also created by fusing PCR-amplified P_*pta-ack*_ promoter into the pMTL84211 vector with no other genes. All subcloning procedures were performed using fusion cloning strategy using NEBuilder HiFi DNA Assembly Kit (New England Biolabs, USA). Transformation was performed according to an earlier described procedure (Westphal et al. 40). In both the plasmids, the butyrate synthetic genes were under the direct control of the strong P_*pta-ack*_ promoter. The plasmids were used to electro-transform *A. woodii* WT cells to generate Aw_ctrl (control), Aw_But6 (6 gene variant), and Aw_But7 (7 gene variant) strains, respectively. The transformants were grown in a volume of 5 ml of carbonate-buffered complex medium (Heise et al. 1989) containing 20 mM fructose and 15 µg/ml erythromycin.

### Semi-quantitative PCR for gene expression analysis

To analyze transcript levels of butyrate synthetic genes in *A. woodii*, RNA was prepared from recombinant *A. woodii* strains (Aw_But6 or Aw_But7) grown on fructose or H_2_ + CO_2_ to mid‐exponential growth phase as described earlier (Chowdhury et al. 2020). A total of 1 μg of RNA from each sample was converted into cDNA by using M‐MLV Reverse Transcriptase according to the manufacturer’s protocol (Promega, Mannheim, Germany). Transcript levels of representative genes of the butyrate operon (*thlA* and *hbd*) and *ptb* were analyzed using gene-specific primers (see Supplementary, Table 1). PCRs were performed using Phusion DNA polymerase (NEB, USA) with 10 ng cDNA as template and 500 nM of gene-specific primers in a final reaction volume of 25 μl. Confirmation of gene expression on different substrates was done by analyzing PCR products on an agarose gel.

### Preparation of cell suspensions and analysis

For cell suspension analysis, recombinant *A. woodii* strains were adapted on either fructose, methanol or H_2_ + CO_2_ and cells were grown in 500/1000 ml volumes to mid-exponential growth phase. Cells were harvested by centrifugation (10,000 × *g*; 10 min) and washed two times with imidazole buffer A (50 mM imidazole–HCl, 20 mM MgSO_4_, 20 mM KCl, 2 mM dithioerythritol (DTE), 1 mg ∙ l^−1^ resazurin, pH 7.0) under strictly anoxic conditions in an anaerobic chamber (Coy Laboratory Products, Grass Lake, MI) filled with 95–98% N_2_ and 2–5% H_2_ as described previously (Heise et al. 1992). Cells were resuspended in 115-ml glass bottles in resuspension buffer (imidazole buffer supplemented with 20 mM NaCl and 60 mM KHCO_3_, pH 7.2) either under a N_2_/CO_2_ or H_2_/CO_2_ atmosphere (80:20 [v/v]).

For determination of the conversion of methanol in cell suspension experiments, 10% CO was added to the N_2_/CO_2_ headspace with no overpressure. For acetogenesis from H_2_ + CO_2_ by recombinant *A. woodii*, a cell concentration corresponding to 1 mg total cell protein per ml and a gas atmosphere of H_2_ + CO_2_ (80:20 [v/v]) at 1 bar overpressure were used. The suspensions were incubated at 30°C in a shaking water bath and substrate/product analyses were done as earlier mentioned. Substrate/product analyses were done from 500 µl samples withdrawn with a syringe at different time points. The concentrations of acetate or butyrate were determined by gas chromatography as described previously (Litty and Müller 2021). The peak areas were proportional to the concentration of each substance and calibrated with standard curves. A total of 5 mM isopropanol was used as the internal standard for all measurements.

### Determination of acetate and butyrate kinase activities

*A. woodii* and *E*. *callanderi* were grown on 20 mM fructose to mid-exponential growth phase, harvested, and washed twice in buffer A (50 mM Tris/HCl, 5 mM MgCl_2_, pH 7.5). Cells were broken in a French pressure cell at 110 MPa and the resulting cell-free extract was freed of cells by low speed centrifugation (8 min, 8000 Upm; JA 25.50 rotor, Beckmann Coulter, Krefeld, Germany). Acetate kinase was measured by an NADH-coupled enzyme assay as described previously (Lindley et al. 1987). The assay mixture contained Tris/HCl (pH 7.5), 4 mM ATP, 1.7 mM PEP, 0.3 mM NADH, pyruvate kinase (1 U), lactate dehydrogenase (1 U), and 750 mM of acetate. The reactions were started with acetate after addition of acetate kinase-containing samples. NADH oxidation was followed at 340 nm. The same assay was used to determine butyrate kinase activity by omitting acetate and adding butyrate (750 mM) instead.

## Supplementary Information

Below is the link to the electronic supplementary material.

Supplementary file1 (DOCX 27 KB)

## Data Availability

Not applicable.
