# Supplementary material for: Biosynthesis of butyrate from methanol and carbon monoxide by recombinant Acetobacterium woodii
Source: Int Microbiol. 2022 Feb 18;25(3):551–60. doi: 10.1007/s10123-022-00234-z (PMC9307552; doi:10.1007/s10123-022-00234-z)
Supplement: Supplementary file 1 — Supplementary file1 (DOCX 27 KB) [file 10123_2022_234_MOESM1_ESM.docx]

**Supporting Information to**

**Biosynthesis of butyrate from methanol and carbon monoxide by recombinant *Acetobacterium woodii***

**Nilanjan Pal Chowdhury** **and Volker Müller^*^**

*Department of Molecular Microbiology & Bioenergetics, Institute of Molecular Biosciences, Johann Wolfgang Goethe University Frankfurt/Main, Max-von-Laue-Str. 9, 60438 Frankfurt, Germany*

Primers used in this study.

Supporting Information Table 1

| Primer Name | Primer Sequence | Target gene  Supplementary S3 |
| --- | --- | --- |
| Elim 0537FqPCR | ACGCTATGGCTACAGAATGG | *Thiolase* |
| Elim 0537RqPCR | GCTTTACGGCTTGAGAAGATTG |  |
| Elim 0538FqPCR | GACCATTCGCTGCCTGATTA | *Hydroxybutyryl-CoA dehydrogenase* |
| Elim 0538RqPCR | GCGTCGTCCATACCCATTT |  |
| Elim 0834FqPCR | AACCAGATAGTTTTCGCTCCTTCGTTTTATTTTT | *Phosphobutyryl transferase* |
| Elim 0834RqPCR | CAGGCCTCGAGATCTCCATGGTTAGTCATACAGGGTTCCCAGC |  |
| 6kb_For Ack | GTGTTAAATTTAAAGGGAGGACGGATCCGTGGCAAAAGAAGTAGTATTAG | *ELI0537-0542 forward primer* |
| 6Kb_Rev_Ack | TTCACTACAAAGTAACATTCCCTAACTCTATCTGGTTGCCATTTC | *ELI0537-0542 reverse primer* |
| pPtaAckfFor_6kb | GATAAAATATTT CTAGACGAGCACTTTCAATATGATATT | P*_pta-ack_ For* |
| pPtaAck Rev_6kb | ACTACTTCTTTTGCCACGGATCCGTCCTCCCTTTAAATTTAACACAAAATT | P*_pta-ack_ Rev* |
| 0834 For_AwBut7 | GAAATGGCAACCAGATAGTTTTCGCTCCTTCGTTTTA | *ELI0834 for* |
| 0834Rev_AwBut7 | CACTACAAAGTAACATTCCCTAACTTTAGTCATACAGGGTTCCC | *ELI0834 Rev* |
